# Obesity and Post-Transplant Diabetes Mellitus in Kidney Transplantation

**DOI:** 10.3390/jcm10112497

**Published:** 2021-06-05

**Authors:** Paloma Leticia Martin-Moreno, Ho-Sik Shin, Anil Chandraker

**Affiliations:** 1 Department of Nephrology, Clinica Universidad de Navarra, Navarra Institute for Health Research (IdiSNA), 31008 Pamplona, Spain; 2 Renal Division, Department of Internal Medicine, Gospel Hospital, Kosin University, Busan 49267, Korea; 67920@naver.com; 3 Transplantation Research Institute, Kosin University College of Medicine, Busan 49367, Korea; 4 Renal Division, Brigham and Women’s Hospital, Harvard Medical School, Boston, MA 02115, USA; achandraker@bwh.harvard.edu

**Keywords:** obesity, kidney transplantation, metabolic syndrome, diabetes mellitus, PTDM, survival, cardiovascular risk, SGLT-2 inhibitors, GLT-1 receptor agonists

## Abstract

Worldwide, the prevalence obesity, diabetes, and chronic kidney disease is increasing apace. The relationship between obesity and chronic kidney disease is multidimensional, especially when diabetes is also considered. The optimal treatment of patients with chronic kidney disease includes the need to consider weight loss as part of the treatment. The exact relationship between obesity and kidney function before and after transplantation is not as clear as previously imagined. Historically, patients with obesity had worse outcomes following kidney transplantation and weight loss before surgery was encouraged. However, recent studies have found less of a correlation between obesity and transplant outcomes. Transplantation itself is also a risk factor for developing diabetes, a condition known as post-transplant diabetes mellitus, and is related to the use of immunosuppressive medications and weight gain following transplantation. Newer classes of anti-diabetic medications, namely SGLT-2 inhibitors and GLP-1 agonists, are increasingly being recognized, not only for their ability to control diabetes, but also for their cardio and renoprotective effects. This article reviews the current state of knowledge on the management of obesity and post-transplant diabetes mellitus for kidney transplant patients.

## 1. Introduction

Obesity, defined by the World Health Organization as a body mass index (BMI) of ≥30 kg/m^2^, is an emerging public health problem [1]. Central obesity is considered an essential component of the metabolic syndrome, according to the International Diabetes Federation criteria [2], at the same time being a modifiable risk factor for the onset and progression of chronic kidney disease (CKD) [3]. Obesity has also traditionally been considered a relative barrier to kidney transplantation [4] and may influence patient survival when it persists or develops after transplantation [5]. In addition, obesity plays a role in the appearance of metabolic syndrome and, especially, post-transplant diabetes mellitus (PTDM) after kidney transplantation [6]. This form of diabetes increases the mortality of kidney transplant patients [7]. Novel treatment options for diabetes mellitus, such as sodium-glucose cotransporter 2 (SGLT-2) inhibitors and glucagon-like peptide 1 receptor (GLP-1) agonists, that have been shown to have cardio and renoprotective effects in non-transplant patients [8], may have an important role to play in the treatment of patients following kidney transplantation.

In this review, we study the importance of pre- and post-kidney transplantation obesity, as well as PTDM. Furthermore, we analyze the different therapeutic approaches currently available, including the new anti-diabetic drugs and their role in the treatment of obesity.

## 2. Obesity before Kidney Transplantation

### 2.1. Epidemiology of Obesity in Patients with End-Stage Kidney Disease (ESRD)

Obesity is currently considered an important preventable risk factor for CKD. In the United States (U.S.), it is estimated that if it were possible to move individuals from the overweight and obese categories to ideal body weight, kidney disease could be prevented in 24.2% of men and 33.9% of women [3].

As with the trend for the general population, the incidence of obesity is also increasing in the dialysis population. Between 1995 and 2002, an increase in the BMI of those patients with ESRD was observed in the U.S. (from 25.7 to 27.5 kg/m^2^), with a rate of BMI change two-times higher than that estimated in the general population for all age groups [9]. The increased prevalence of obesity in the ESRD population reflects the risk of obesity for CKD [10].

The burden of CKD is increasing. According to the U.S. Renal Data System, the prevalence of CKD was 12.3% from 1988 to 1994 and increased to 14% between 2005 and 2010. In a historical cohort that included 320,252 adults, with a total of 1471 cases of ESRD, elevated BMI remained an independent predictor for ESRD after adjusting for confounding parameters, such as blood pressure level and presence or absence of diabetes mellitus [11].

It should also be noted that while BMI is almost universally used to study obesity, it may not be the best tool for understanding the adverse effects of obesity in all populations [12]. Indeed, waist circumference is highly correlated with the amount of intra-abdominal fat and has been shown to be more related to cardiovascular disease risk factors than BMI [13]. Additionally, although assessment of body composition with a device, e.g., air-displacement plethysmography (BodPod), is not required for the management of obesity, it may be useful for measuring fat and lean mass [14].

### 2.2. Morbidity and Mortality in Obese ESRD Patients

The wait time for transplantation in obese patients with ESRD is increasing, which is compounded by increasing morbidity while on the transplant waiting list compared to those transplanted [15,16].

These patients may be excluded from transplantation due to concerns about reduced graft survival and increased risk of side effects, including patient survival after transplantation, owing to the perception that outcomes are worse in patients with ESRD and a high BMI (>30 kg/m^2^) [5,17,18].

However, data obtained by the UK National Health Service Blood and Transplant Registry showed that individuals transplanted during 2004–2010 had a significantly lower risk of death compared to those who remained on the waiting list in all BMI groups, including the >35 BMI group [16].

Despite this, an obesity paradox in patients on dialysis has been observed, whereby a high BMI is associated with better survival, complicating the issue of how much weight loss should be recommended prior to transplantation [19]. It is also important to consider that if obesity is associated with type 2 diabetes mellitus, the survival rate described is lower [20].

### 2.3. Risks of and Contraindications for Kidney Transplantation

BMI-based cutoffs have been used as part of candidate selection for transplantation in most centers [21]. Results from a worldwide survey of 399 nephrologists indicated a reluctance to recommend patients with a BMI higher than 30 or 35 kg/m^2^ for kidney transplantation, and instead suggested weight loss programs prior to referral [4,22].

To optimize post-transplant outcomes, the existing clinical practice guidelines of the American Society of Transplantation and the European Renal Association recommend targeting a pre-transplant BMI of <30 kg/m^2^ with diet and exercise under supervision [23,24]. However, the KDIGO guidelines suggest that transplant candidates should not be excluded from transplantation because of obesity, although they ought to be offered weight-loss interventions prior to transplantation [25]. Therefore, recommendations on weight loss before receiving a kidney transplant remain controversial, as there is no evidence of improved long-term post-transplant outcomes, and that significant weight loss appeared to be transitory [26]. An older study by Modlin et al. found that cardiac disease was the main cause of death in obese patients, proposing that it may be particularly important to set weight targets for obese patients with a history of ischemic heart disease, prior to consideration for kidney transplantation [27].

### 2.4. Treatment Options for Overweight in Patients with ESRD

#### 2.4.1. Lifestyle Interventions and Medical Treatment

A very low-calorie diet is difficult to follow in the long-term, especially in dialysis patients, given the dietary restrictions for CKD and the high protein intake recommended for these patients. Regular physical activity level should therefore always be considered as part of weight loss management [28], however regular physical activity for hemodialysis patients is not always possible because of dialysis associated fatigue.

A study including 80 patients with stage 4–5 CKD and a BMI > 35 kg/m^2^, in a weight loss program based on healthy eating habits (maintaining pre-established dietary restrictions) and higher energy expenditure, showed a modest effect only when a mild weight loss was required. The main problem described was the length of time required to reach weight loss goals, along with a frequent rebound weight gain effect [29].

Unfortunately, most drugs utilized for weight loss (e.g., phentermine, diethylpropion, sibutramine, fluoxetine (high dose), and sertraline (high dose)) are contraindicated for patients on dialysis [30].

#### 2.4.2. Bariatric Surgery

Lafranca et al. recommended that obese kidney transplant candidates with a BMI > 40 kg/m^2^, or >35 kg/m^2^ plus associated comorbidities, should be considered for bariatric surgery [31]. This procedure has historically been considered as high risk for kidney transplant candidates, however it has recently been shown to have acceptable morbidity and mortality in ESRD patients [32].

Several recent studies have reported successful transplant performance after bariatric surgery. Many of these are series of studies in which 55% to 100% of the patients undergoing bariatric surgery either receive a kidney transplant during follow-up or are successfully listed for transplantation [33]. Side effects specially related to management of immunosuppressive medications should be considered, as shown in Table 1 [34].

## 3. Obesity and PTDM after Kidney Transplantation

### 3.1. Epidemiology of Obesity after Kidney Transplantation

The prevalence of obesity among kidney transplant recipients in the U.S. had increased to over 30% (10% with morbid obesity) in 2011, whereas the obesity rate in the 1990s was less than 20% [15,35]. In comparison, in one study concerning the transplant population in Australia and New Zealand, 54% of adult kidney recipients were overweight or obese [36].

Obesity is also a frequent complication that develops following kidney transplantation, especially during the first year. In a retrospective study conducted in Brazil involving 374 patients, 31.2% and 20.9% gained at least 10% or 5% of baseline weight, respectively, after the first year of transplantation [37]. According to Clunk et al., the main factors influencing weight gain after kidney transplantation were female sex, low median household income, and younger age [38].

### 3.2. Post-Transplant Diabetes Mellitus (PTDM)

The term PTDM defines clinically stable patients who develop persistent post-transplant hyperglycemia [39]. Its incidence ranges from 2% to 53% [7]. Although the terminology New Onset Diabetes Mellitus (NODAT) has been commonly used, an International Expert Panel of clinicians and researchers recommended the term PTDM because it better describes newly diagnosed diabetes mellitus in the post-transplant period (irrespective of whether it was present but it had not been detected before transplantation) [39].

PTDM is usually diagnosed at least 45 days post-transplant, using the American Diabetes Association (ADA) criteria for type 2 diabetes mellitus with the 75 g OGTT, or with alternative criteria of fasting plasma glucose ≥ 126 mg/dL, or symptomatic hyperglycemia with a plasma glucose ≥ 200 mg/dL, or a glycated hemoglobin (HbA1c) ≥ 6.5% [40,41].

### 3.3. Pathophysiology and Risk Factors for PTDM

Although PTDM shares features with type 2 diabetes mellitus, such as insulin resistance and obesity, the underlying processes are thought to be more complex [42]. In general, the pathophysiological mechanisms responsible for PTDM include lower number and binding affinity of insulin receptors, impaired glucose absorption in peripheral organs, and glucose/fatty acid pathway activation. These mechanisms are particularly important for patients with significant weight gain after transplantation [43]. In addition, concurrent with reduced insulin secretion, an imbalance in the insulin-glucagon axis during hyperglycemia with a reduced glucose-induced glucagon suppression has been described in a study that included Caucasian kidney transplant patients with PTDM. In the same study, Halden et al. showed that administration of a GLP-1 infusion, an incretin hormone responsible for the insulin response after glucose ingestion, caused markedly lower glucagon responses, along with higher insulin responses, compared to saline [44].

The risk factors for developing PTDM (Table 2) include, among others, increasing age, obesity, viral infections, adverse effects of immunosuppressive drugs, and hypomagnesemia [41,45].

In the context of obesity, an increased fat mass with adipose tissue dysfunction, characterized by altered lipid storage capacity and low-grade inflammation, favors the development of insulin resistance and impaired glucose metabolism by promoting an excess of fat storage in non-adipose tissues, such as the pancreas [50].

Regarding viral infections, a higher incidence of PTDM with cytomegalovirus infections and hepatitis C has been reported. In the case of cytomegalovirus, one described mechanism may be due to impaired insulin release [51]. Hepatitis C infection has been shown to be associated with insulin resistance. A recent study that included liver transplant recipients showed that the eradication of hepatitis C virus with highly effective direct acting antiviral therapy is associated with a lower incidence of PTDM [52].

Hypomagnesemia was reported to be an independent risk factor for PTDM in a retrospective study of 948 kidney transplant patients. Magnesium is an intracellular cofactor necessary for different processes such as intermembrane glucose transport and glucose oxidation. Additionally, the use of certain drugs can cause hypomagnesemia [46].

Finally, increased odds of developing PTDM among kidney transplant recipients with autosomal dominant polycystic kidney disease (ADPKD) have also been described [53], potentially related to polycystin-1 and polycystin-2 expression in organs outside of the kidney, such as the pancreas [54].

### 3.4. Effects of Different Immunosuppressants on PTDM

Glucocorticoids cause hyperglycemia by increasing glucose resistance, decreasing insulin secretion, inducing beta cell apoptosis, and reducing the expression of glucose transporters [55]. A large retrospective study of more than 25,000 transplant recipients showed that steroid-free immunosuppression was associated with a lower risk of PTDM occurrence compared to steroid-containing therapy. The cumulative incidence of PTDM within 3 years of transplantation was 12.3% and 17.7% for steroid-free therapy and steroid-containing therapy, respectively [53]. However, in a recent double-blind 5-year study comparing a group of patients who initially stopped corticosteroids with a group who reduced corticosteroids to 5 mg/day after 6 months, there was no difference seen in the rate of PTDM (35.9% in the group that discontinued corticosteroids, and 36.3% in the group that maintained 5 mg per day after 6 months) [56].

Calcineurin inhibitors (CNIs), both cyclosporine and tacrolimus, increase the risk of PTDM by reducing insulin secretion with a direct toxic effect on pancreatic beta cells. This effect has been described to be more significant and in a dose-dependent manner, with tacrolimus than with cyclosporine [57]. In fact, according to the DIRECT study, the incidence of PTDM 6 months after transplantation was significantly lower in patients treated with cyclosporine than in tacrolimus-treated patients [58]. It is also interesting to note that calcineurin inhibitors cause hypomagnesemia, which as mentioned previously increases the risk of developing PTDM [59].

Concerning the different tacrolimus formulations, a study including 133 patients showed no differences in blood glucose levels two years after conversion from tacrolimus twice-daily to the once-daily extended-release tacrolimus formulation [60]. Furthermore, Voclosporin, a novel calcineurin inhibitor, does not seem to cause the same level of inhibition of insulin secretion as tacrolimus does, which may be related to a lower incidence of PTDM [61,62].

M-TOR inhibitors may influence the PTDM development through different mechanisms, such as impaired insulin secretion and reduced insulin signal transduction [63]. In one study, the use of an m-TOR (sirolimus) in combination with a CNI (cyclosporine or tacrolimus), resulted in the highest incidence of PTDM [43]. However, in a recent work, the combination of everolimus with low-dose tacrolimus, compared to standard-dose tacrolimus, showed no difference in PTDM rates [64].

Other immunosuppressants, including azathioprine and mycophenolate mofetil (MMF), have not been shown to induce diabetes. The combination of tacrolimus and MMF or cyclosporine and MMF was found to have a lower PTDM rate than tacrolimus and azathioprine [44].

Moreover, a review of five studies, which included 1535 kidney transplant recipient, concluded that belatacept-treated kidney transplant recipients had lower PTDM rates compared to those who received calcineurin inhibitors [65].

Therapeutic options to prevent the risk of PTDM may involve reduced doses of steroids, minimization of tacrolimus exposure, and conversion from tacrolimus to belatacept, although the risk for rejection must be considered. We will have to wait until new immunosuppressive strategies, such as the use of an anti-CD40 monoclonal antibody that has shown efficacy in animal models of transplantation, with a significant reduction in the onset of primary autoimmune diabetes, can be used in humans [66,67].

## 4. Morbidity and Mortality in Patients with Obesity after Kidney Transplantation 

### 4.1. Patient and Graft Survival

BMI has been shown to be a strong independent risk factor for patient mortality and graft failure. Meier-Kriesche et al. demonstrated in a study of 51,927 kidney transplant recipients that the association between BMI and mortality after kidney transplantation follows a U-shaped pattern, with the lowest risk observed in patients with a BMI between 22 and 32 kg/m^2^. The authors also found that increased BMI is a risk factor for death-censored graft survival, although they were not able to assess whether the risk is mediated by a higher incidence of comorbidities, such as diabetes and hypertension, or by factors intrinsic to obesity like hyperfiltration [5]. Indeed, PTDM has been associated with lower patient survival rates [68].

Data from a French cohort including 4691 kidney transplant patients, 747 of whom were obese, also showed an increased risk of death in obese patients compared to non-obese subjects [69].

However, a meta-analysis demonstrated that obesity in kidney recipients who were transplanted after the year 2000 is not associated with graft failure or greater risk of death [45], although morbid obesity (BMI ≥ 35 kg/m^2^) has been consistently associated with an increase in the risk of death and graft loss [5,17].

Finally, a study showed that BMI and waist circumference were independent predictors of albuminuria, a predictor of allograft failure, cardiovascular disease, and mortality [70], in the late post-transplant period [71].

### 4.2. Surgical and Other Complications

Historically, obese kidney transplant recipients have tended to have a higher risk of delayed transplant function, wound complications, surgical site infection, and long-term hospitalization [69]. Thus, obesity has become a major economic and health burden for kidney transplantation, and early studies have shown that post-transplant side effects increase as BMI increases [5,17,46], although its relevance in recent studies was not so clear [47].

It has been previously reported that the relative risk of delayed allograft function increases progressively with increasing BMI, being the highest risk in those with a BMI > 36 kg/m^2^ [5].

In a series of 2013 transplant recipients, it was found that the most significant risk factor for a wound infection was obesity (BMI > 30 kg/m^2^), while age and obesity were significant risk factors for hernia [48]. A study that compared the use of mycophenolate mofetil with sirolimus on surgical complications described that 38% of those with a BMI > 30 kg/m^2^ suffered wound complications vs. only 20% of those with a BMI < 30 kg/m^2^ (*p* = 0.029) [72]. Robot-assisted kidney transplantation may become the preferred technique for transplanting morbidly obese recipients, although its availability remains extremely limited, and abandoning strategies targeting weight loss pre-transplantation would be premature [73].

Finally, in a meta-analysis that included 21 retrospective observational studies with a patient sample size of 241,381, obese recipients showed a higher risk of having biopsy-proven acute rejection and delayed graft function, compared with their healthy BMI counterparts [49].

### 4.3. Cardiovascular Outcomes

In one study in the BMI group > 36 kg/m^2^, there was an exponential increase in the relative risk for cardiovascular death [5], which has also been described to be the main cause of death with functioning graft between kidney transplant patients [52]. Worse outcome differences, described by Modlin et al. in 127 obese kidney transplant patients (BMI > 30 kg/m^2^) compared with a matched non-obese control group (BMI < 27 kg/m^2^), were primarily due to a higher mortality resulting from cardiac events [27]. In the French cohort, obese kidney transplant patients were also at a higher risk of cardiac complications compared to non-obese patients [69].

In a study that included more than 700 kidney transplant patients, the majority treated with tacrolimus, mycophenolate, and prednisone, pretransplant obesity was one of the factors that affected early and late PTDM, its reversibility, and its evolution from prediabetes to PTDM [74].

Furthermore, in a retrospective study including 650 nondiabetic, nor obese patients, without documented ischemic heart disease, who received living-donor kidney transplants, the development of obesity at 6 months post-transplantation was associated with an increased risk of PTDM, hypertension, hyperlipidemia, and decreased patient survival at 5 and 10 years, probably due to greater cardiac morbidity [75].

Regarding PTDM itself, Wauters et al. showed that PTDM increased cardiovascular risk and major cardiovascular events [76].

## 5. Treatments for Obesity and PTDM in Kidney Transplant Patients

In a study conducted in a large prospective cohort of 817 kidney transplant recipients, it was shown that increased daily-life moderate-to-vigorous physical activity was associated with a reduced risk of PTMD, as well as cardiovascular and all-cause mortality, although the association with PTDM was not significant after being adjusted for metabolic confounding factors, such as glucose level. The authors propose embedding physical activity in the health care management of kidney transplant patients [77].

Concerning lifestyle interventions for the management of obesity and PTDM in kidney transplant recipients, the joint position statement of the Italian Societies of Nephrology Organ Transplantation and Diabetes, suggest to follow the nutritional recommendations developed for the general population with diabetes [78,79]. They also recommend encouraging physical exercise and, in overweight/obese patients, weight loss by offering a weight-reduction program or bariatric surgery if required [79]. In a study comparing 22 morbidly obese kidney transplant patients who underwent bariatric surgery, with 44 moderate to severely obese patients on lifestyle management, significant reductions in mean BMI at 6 months were observed only in the group with bariatric surgery. However, the mean tacrolimus dose was significantly lower in the non-bariatric group, reflecting lower tacrolimus absorption in the bariatric group [80].

In addition to insulin treatment, anti-diabetic drugs for PTDM such as metformin, sulfonylureas, meglitinides, and dipeptidyl peptidase IV inhibitors (DPP4-i) have been used in kidney transplant patients according to patient characteristics and renal function as shown in Table 3 [41,79] with no substantial interaction being seen in terms of the pharmacokinetics of tacrolimus, cyclosporine, or m-TOR inhibitors [42]. In a study that included 14,144 kidney transplant patients with type 2 diabetes mellitus, low risk for all-cause mortality was described in regimens that included metformin compared with those with insulin [81], something similar to what had been described in the general population [82]. However, the use of metformin, which inhibits the liver mitochondrial glycerophosphate dehydrogenase involved in gluconeogenesis, is limited due to the risk for lactic acidosis in advanced CKD, so its use in kidney transplant patients is uncommon [81]. The use of DPP4-i, a group of drugs that stabilize the GLP-1 hormones, has been shown to be safe and more effective that sulfonylureas in improving glycemic control in kidney transplant patients, but differences in body weight was not observed between both treatments [83]. Finally, the combination of metformin and the DPP4-i sitagliptin have demonstrated greater weight reductions than the combination of metformine and insulin glargine in kidney transplant patients [84].

Recent studies have shown the advantages of sodium-glucose cotransporter 2 (SGLT-2) inhibitors and glucagon-like peptide 1 receptor (GLP-1) agonists on cardiac and renal outcomes in non-transplant patients.

SGLT-2 inhibitors are novel oral glucose-lowering medications that inhibit glucose and sodium reabsorption in the proximal tubule. As a result of the increased natriuresis and concurrent osmotic diuresis due to its glycosuric effects, SGLT-2 inhibitors decrease blood pressure and increase hematocrit [86]. However, the importance of these drugs is related to reductions in cardiovascular risk, as well as the strong reductions in risk for heart failure that was demonstrated in three cardiovascular outcome trials [87,88,89]. In addition, SGLT-2 inhibitors have shown efficacy in decreasing albuminuria, even in patients using renin-angiotensin system blockade therapy, suggesting they confer additional renoprotective effects [59]. Finally, the DAPA-CKD trial concluded that patients with CKD, independent of the presence or absence of type 2 diabetes, who received dapagliflozin, had a significantly lower risk of a composite of a sustained decline in the estimated GFR of at least 50%, ESRD, or death from renal or cardiovascular causes than those who received a placebo [90].

With respect to the use of SGLT-2 inhibitors in kidney transplant patients, a meta-analysis that included eight studies with a total of 132 patients, one randomized controlled trial and seven case series, demonstrated that SGLT-2 inhibitors effectively lowered HbA1c when patients were treated for at least 12 months, and reduced body weight when treated for at least 6 months. Serum creatinine levels and urine protein-creatinine ratio were stable throughout the follow-up period. Moreover, canagliflozin showed efficacy in diminishing systolic blood pressure. Adverse effects of SGLT-2 inhibitors included urinary tract infection (43.8%), small ulcers in lower extremities (10%), cellulitis (10%), acute kidney injury (3.6%), and genital fungal infection (1.4%), but no acute rejection events were reported [91]. In the randomized controlled trial that included 22 kidney transplant recipients treated with empagliflozin, compared to 22 with placebo, reduced glucose-lowering effect was related to lower GFR at baseline [92].

The incretins glucagon-like peptide 1 (GLP-1) are gut-derived hormones that potentiate insulin secretion and contribute to glucose metabolism. As GLP-1 actions are reduced in type 2 diabetes mellitus, several GLP-1 receptor agonist formulations have been introduced for glucose lowering in this type of diabetes. Additionally, GLP-1 receptor agonists lead to reduced body weight, blood pressure, and lipid levels [93], and when compared to insulin showed a reduced decline in estimated GFR [94]. With respect to its effects in the reduction in body weight by inhibit feeding, it has been suggested that in the near future the efficacy gap between these drugs and bariatric surgery will narrow [95], perhaps making GLP-1 receptor agonists the treatment of choice for obese patients.

In a study that included kidney transplant patients, exogenously delivered GLP-1 improved insulin secretion and glucose-induced glucagon suppression in a hyperglycemic state [44]. With respect to the clinical experience, there are only retrospective studies regarding the use of GLP-1 receptor agonists in transplant patients that describe a significant decrease in weight and insulin requirement [96] without serious adverse defects, gastrointestinal-related being the most frequent, and without significant changes in tacrolimus level [97,98]. Finally, in a retrospective study that included solid organ transplant patients with type 2 diabetes mellitus (diagnosed pre- or post-transplant) and stage 3–4 of CKD in more than 70% of the patients, dulaglutide compared with liraglutide showed a trend towards improved kidney function over a 24-month follow-up period [96].

The ADA and European Association for the Study of Diabetes (EASD) Consensus Report recommended GLP-1 receptor agonists or SGLT-2 inhibitors for type 2 diabetics with established atherosclerotic cardiovascular disease or CKD, if their HbA1c levels were above 7% under therapy with metformin [99]. More recently, the consensus statement of the EURECA-m and the DIABESITY working groups of the ERA-EDTA recommend that GLP-1 receptor agonists should be used in patients with type 2 diabetes mellitus and CKD immediately after SGLT-2 inhibitors to maximize cardio and renoprotection [8]. According to those recommendations, additional randomized controlled trials, longitudinal observational studies, and multicenter studies investigating the use of SGLT-2 inhibitors and GLP-1 agonists in kidney transplant recipients with, and perhaps without diabetes mellitus, but with other cardiovascular risk factors such as obesity, are urgently needed.

## 6. Conclusions

While superficially it may seem that weight loss can only be good in patients with CKD and post-transplantation, the area remains quite challenging. Restrictive diets, the high prevalence of diabetes, the difficulty of maintaining an active lifestyle, weight loss goals, as well as the potential side effects of medications and bariatric surgery all compound the difficulty of managing this patient population. Despite this, newer medications such as SGLT-2 inhibitors and GLP-1 agonists may offer much needed novel therapeutic approaches to weight loss, and need to be better studied in the CKD, ESRD, and transplant populations.

## Figures and Tables

**Table 1:** Effect on Drug levels of bariatric surgeries [34].

Type of Surgery	Effect on Drug Levels
Gastric bypass	Higher doses of immunosuppressive medications
Biliopancreatic diversion	Not reported
Laparoscopic gastric banding	None reported
Vertical gastroplasty	None reported

**Table 2:** Risk factors for PTDM [41,46,47,48,49].

Risk factors	Relative Risk for PTDM (95% CI)	*p*-Value
Old age (≥60)	2.60 (2.32–2.92)	<0.0001
African-American race	1.68 (1.52–1.85)	<0.0001
Hispanic ethnicity	1.35 (1.19–1.54)	<0.0001
Obesity	1.73 (1.57–1.90)	<0.0001
Family history of DM	3.14 (1.87–5.27)	<0.0001
Steroid-containing maintenance regimen	1.42 (1.27–1.58)	<0.001
Tacrolimus	1.53 (1.29–1.81)	<0.0001
HCV positive	1.33 (1.15–1.55)	<0.0001
Hypomagnesemia	1.58 (1.07–2.34)	0.02
HLA mismatches (6)	1.30 (1.07–1.58)	0.0085
Donor characteristics (male)	1.12 (1.03–1.21)	0.0090

Abbreviations: DM, diabetes mellitus; HCV, hepatitis C virus; HLA, human leukocyte antigen.

**Table 3:** Therapeutic groups for PTDM according to advantages and disadvantages [85].

Type of Anti-Diabetic Drug	Advantages	Disavantages
Insulin	No dose adjustment in renal impairment	Hypoglycaemia Weight gain Injectable
Metformin	No hypoglycaemia No weight gain	Contraindicated if eGFR < 30 mL/min/1.73 m^2^ Caution if eGFR 30–59 mL/min/1.73 m^2^ Gastrointestinal side effects, Risk of lactic acidosis
Sulfonylurea/glinides	Rapid onset of action	Hypoglycaemia Weight gain Increased CV risk Dose adjustment required in renal impairment
Thiazolidinediones	No dose adjustment in renal impairment	Weight gain Increased fracture risk Potential risk for CHF
DPP4-i	No hypoglycaemia No weight gain	All but linagliptin require dose adjustment in renal impairment Potential risk for CHF (saxagliptin, alogliptin)

Abbreviations: eGFR, estimated glomerular filtration rate; e, congestive heart failure; and DPP4-I, dipeptidyl peptidase-4 inhibitors.

## Data Availability

No new data were created or analyzed in this study. Data sharing is not applicable to this article.
